# The relationship between the interactive behavior of industry–university–research subjects and the cooperative innovation performance: The mediating role of knowledge absorptive capacity

**DOI:** 10.3389/fpsyg.2022.1077614

**Published:** 2023-01-05

**Authors:** Xiulan Meng, Kui Di, Huan Su, Xiaoyan Jin, Weiwei Lv, Xingqi Huang, Chaoyang Wu, Li Fan

**Affiliations:** ^1^Xingzhi College of Zhejiang Normal University, Lanxi, Zhejiang, China; ^2^Jinhua Polytech, Coll Informat Engn, Jinhua, Zhejiang, China; ^3^Modern Management College of Zhejiang Industry and Trade Polytechnic, Wenzhou, Zhejiang, China; ^4^Accounting Department, Zhejiang University, Shanghai College of Finance and Economics, Jinhua, Zhejiang, China; ^5^Zhejiang Gongshang University College of Management Engineering and E-Commerce (Cross Border E-Commerce College), Hangzhou, Zhejiang, China

**Keywords:** industry–university–research cooperation, cooperative trust, cooperative communication, knowledge absorptive capacity, cooperative innovation performance

## Abstract

**Introduction:**

Industry–university–research cooperation innovation, which is often characterized by resource complementarity and the sharing technology, has become one of the most preferred innovation cooperation methods for enterprises. However, various problems still occur in the process of industry–university–research cooperations, such as poor innovation performance and difficulty in sustaining cooperation. Existing studies mostly focus on the macroscopic perspectives of geographic location, cooperation scale, concentration, and diversification of industry–university–research cooperation subjects, and fail to explore the microscopic behavioral mechanisms.

**Methods:**

Therefore, this paper establishes the interactive behavior of industry–university–research subjects and defines its concepts and dimensions in an attempt to provide a mechanism for improving the cooperative innovation performance of industry–university–research from the micro-behavioral perspective. On the basis of theoretical analysis, this paper develops a model of the relationship between cooperative trust, cooperative communication, and cooperative innovation performance for interactive behavior, while exploring the mediating role of knowledge absorptive capacity. The model was validated by stepwise regression using data from 325 questionnaires.

**Results:**

The paper found that cooperative trust and cooperative communication in the cooperative interactive behavior of industry–university–research positively contribute to the improvement of cooperative innovation performance. Knowledge absorptive capacity plays a partially mediating role between the interactive behaviors and cooperative innovation performance. More specifically, knowledge absorptive capacity partially mediates cooperative communication in cooperative innovation performance and completely mediates cooperative trust in cooperative innovation performance. The results are largely consistent with the results of the heterogeneity analysis of the sample.

**Discussion:**

This paper not only explains why the cooperative innovation performance of industry–university–research is poor from the perspective of interactive behavior, but also enriches the research perspective of industry–university–research and provides theoretical support for enterprises to optimize the relationship between industry, university, and research institutes.

## Introduction

Strengthening industry–university–research cooperation, making good use of the innovative resources of universities, and promoting knowledge transfer and the transformation of achievements play an important role in accelerating the innovation–driven and high–quality development of China’s economy (Wang et al., 2018). With the introduction of China’s innovation–driven strategy, industry–university–research cooperation has made rapid progress. The enthusiasm of cooperation subjects has increased significantly, cooperative innovation has become increasingly active and the transformation of scientific and technological achievements has been deepened. Industry–university–research cooperation plays an important role in accelerating the innovation–driven and high–quality development of the economy. In recent years, an increasing number of enterprises have chosen to cooperate with universities and research institutes in an attempt to break the innovation chain more rapidly (Wang and Hu, 2022). It has been proven that industry–university–research cooperation is an effective means for enterprises to obtain complementary resources, create new knowledge and develop new technologies, and improve their technological innovation capabilities in the era of open innovation (Ma et al., 2018). According to the 2020 China Enterprise Innovation Capability Statistical Monitoring Report, the proportion of enterprises cooperating with industry, universities, and research institutes was 34.7%, i.e., enterprises conducting innovation cooperation. However, in the process of promoting industry–university–research cooperation, as a result of the information asymmetry of technology supply and demand, low trust in cooperative communication, and the concept of emphasizing results over process deviation, the practical problems of insufficient breadth, insufficient depth, and insufficient performance of industry–university–research cooperation exist. For many years, industry–university–research cooperation has been an important issue in the management of innovation academia (Qing and Qi, 2020; Li et al., 2022), and how to effectively improve the cooperative innovation performance of industry–university–research is an urgent problem to be solved (Cao et al., 2013; Yan et al., 2017; Liu et al., 2020).

The current research results can be summarized as follows. Firstly, focusing on the network structure of industry–university–research cooperation, it is demonstrated that network centrality, structural holes, network location, network density, and other network factors affect the cooperative innovation performance of industry–university–research (Li et al., 2020; Guo et al., 2022; Li and Jian, 2022). Secondly, focusing on the geographical location of industry–university–research subjects, it is shown that the objective geographical environment, including factors such as geographical proximity, geographical distance, and geographical concentration of industry–university–research subjects, affects the innovation performance of industry–university–research (Wang and Luan, 2019; Zhang and Shen, 2019; Zhang and Chen, 2022). Thirdly, focusing on the characteristics of the industry–university–research cooperative subjects themselves, it is indicated that the resource environment, including diversity, heterogeneity, matching, and complementarity of industry–university–research subjects, affects the innovation performance of industry–university–research (Li and Zhu, 2019; Zhang et al., 2021; Yang et al., 2022). Fourth, focusing on the external environment of industry–university–research, it is shown how factors such as the business environment, institutional development stage, market competition, financing environment, and government subsidies affect the innovation performance of industry–university–research (Zhang et al., 2019, 2021, 2022; Song et al., 2022). Fifth, focusing on enterprises’ own capabilities, it is pointed out that the soft environment of enterprises, which includes factors such as knowledge absorptive capacity, knowledge base, and learning capacity, affects the innovation performance of industry–university–research (Lu and Ye, 2017; Li et al., 2020; Zhang et al., 2022). The above research results are relatively rich, and provide explanations for the influence of the macromarket network environment, geographical environment, and resource environment on the cooperative innovation performance of industry–university–research. This also led the authors to ask whether, in addition to the impact of the macroenvironment on the performance of industry–university–research cooperation innovation, whether the microbehavior of the cooperation subject also has an impact on cooperative innovation performance?

On the basis of the shortcomings of existing research, this paper begins to explore the micro-impact mechanisms of the cooperative innovation performance of industry–university–research cooperation. Various scholars have proposed that the role of industry–university–research in encouraging innovation performance in China is not obvious, mainly because the internal subjects of industry–university–research lack long-term and deep interactions (Chen et al., 2009). Through interaction, universities, research institutes, and enterprises form a relationship of interdependence, interaction, mutual promotion, mutual penetration, and mutual restriction, which is the basis of effective cooperation between industry, university, and research (Li and Ye, 2011; Li and Liu, 2013). However, this kind of cooperation is only a static relationship, and its cooperative role and effectiveness must be realized through the interaction between the subjects (Li and Liu, 2013). Li Chenglong first proposed the concept of interactive behavior in industry–university–research cooperation, but unfortunately did not provide a detailed introduction to or explore the essence of interactive behavior. In 2016, Hu Junyan and others divided industry–university–research interaction into low interaction and high interaction, and proposed that interaction intensity has a positive impact on performance (Hu et al., 2016). In addition, the essence of the goal of industry–university–research cooperation is to realize the transfer of knowledge. Industry–university–research cooperation has built channels for knowledge transfer (Jin et al., 2015). However, in actual industry–university–research cooperation, the mismatch between the capabilities of enterprises and universities leads to a structural dilemma, which makes it impossible to effectively internalize external resources and knowledge (Wang et al., 2022), resulting in poor knowledge transfer. The efficiency of knowledge transfer largely depends on the knowledge absorptive capacity of enterprises (Ye and Chen, 2022). Knowledge absorptive capacity not only determines the breadth and depth of knowledge flow, but also promotes the process of knowledge flow (Rupietta and Backes-Gellner, 2017).

At present, the theoretical explanation of the impact of micro behavior on cooperative innovation performance is seriously insufficient. The logical relationship between micro behavior and enterprise knowledge absorptive capacity is relatively unexplored. Wu et al. (2022) pointed out that the trust relationship of industry–university–research cooperation can accelerate the flow of knowledge (Wu et al., 2022). When cooperative organizations have a degree of trust, knowledge is more likely to be transferred. However, knowledge transfer does not mean the improvement of cooperative innovation performance, but increases the possibility of this. Therefore, on the basis of transaction cost theory and a resource-based view, this paper attempts to explore two points. Firstly, we assess interactive behavior of industry–university–research cooperation subjects, and how to affect cooperative innovation performance. Secondly, we explore whether the knowledge absorption capacity of enterprises can improve the cooperative innovation performance of industry–university–research, and what the logical relationship between interactive behavior is composed of. In this paper, our research combined with existing achievements provides a breakthrough from the microperspective in terms of exploring the impact of the behavior characteristics of the cooperation subject on the cooperative performance of industry–university–research, which belongs to the category of organizational behavior.

## Theory and hypotheses

### Interactive behavior of industry–university–research cooperation

The academic community has not yet reached a consensus on the conceptual definition and dimensional division of the interaction behavior of industry–university–research subjects. More research is conducted based on the interaction process, and it is believed that the interaction behavior is mainly expressed through the interaction process, such as through trust, communication, conflict, cohesion, coordination, and learning in the interaction process (Tison and Poirier, 2021; Yang et al., 2022). One of the more representative definitions is Marks’ definition. He thinks that interactions between members and between members and outside subjects constitute interaction processes. Communication and conflict resolution, and communication and trust in interpersonal relationships are frequent interaction activities (Hoegl and Gemuenden, 2001). Li and Liu (2013) defined industry–university–research interaction behavior as the manifestation of the interaction between partners. It is the cognitive, verbal, and behavioral activities that maintain contact and connectivity between partners. It is more reflected in the interaction of various partners to complete the task objectives, emphasizing behavior and process (Li and Liu, 2013). Hu et al. (2016) believe that industry–university–research cooperation is actually a kind of cooperation between heterogeneous organizations, and the process of interaction between cooperative subjects includes knowledge spillover, information exchange, trust, and cohesion.

In 1987, Hackman classified interaction behaviors into two categories: affective interaction behaviors and task-based interaction behaviors. Affective interaction behaviors are interpersonal processes that occur during collaboration, including trust, cohesion, and conflict reduction. Task interaction behaviors are those directly related to the R&D innovation task and include knowledge sharing, communication and balanced member contributions (Hackman, 1987). Judging from the essence of industry–university–research cooperation, industry–university–research comprises realizing the knowledge transfer (Jin et al., 2015). However, as universities and enterprises are in different knowledge positions, there is a conflict in the consistency of cooperative innovation goals, a situation of nonfinite rationality, a high degree of uncertainty, and information asymmetry, which leads an increase in the risk of industry–university–research (Li et al., 2015). The conflict can be effectively managed if the parties can have complete trust in one another throughout the interaction process, which can enhance transaction efficiency and innovation performance and lower transaction costs (Veugelers and Cassiman, 2005). Domestic scholars have also paid attention to the communication behavior in the interaction process. When the cooperative organization has a certain degree of trust, it can strengthen the willingness of the subject to share knowledge. At this time, knowledge is more easily transferred and it is easier to create a good atmosphere for innovation (Wang et al., 2018) In addition, the communication behavior in the process of interaction has also attracted the attention of domestic scholars. Communication is the basis of interaction between universities, research institutes, and enterprises, facilitating the effective transfer of innovative ideas in the collaborative process and making these ideas subject to evaluation by other partners, which, in turn, has a positive impact on innovation performance (Li and Liu, 2013). Therefore, better cooperative communication and integration arrangements are key to enhancing collaborative performance (Bai and Chen, 2022). Judging from the interaction process of industry–university–research cooperation, it also is a game. The cooperative game is similar to the prisoner’s dilemma. To increase the probability of the emergence of the Pareto-optimal Nash equilibrium, the same coordination game can be played repeatedly (Duan et al., 2022). In contrast, the emergence of a Pareto-optimal Nash equilibrium can only be achieved by information communication in the game.

On the basis of the above analysis, it can be judged that trust and communication in the process of industry–university–research cooperation are two very important factors affecting the cooperative innovation performance. Industry–university–research interaction behavior refers to the process of cognitive, linguistic, and behavioral activities that are carried out in order to accomplish the goal of cooperation and to maintain mutual contact, and mutual assistance between universities, research institutes, and enterprises. It is also a manifestation of interaction, emphasizing the behavior and process between subjects. The realization of this process relies on trust and communication between subjects. Therefore, industry–university–research trust and communication are the main microfactors that were revealed in this study. In order to fit the context of industry–university–research cooperation and to distinguish it from trust and communication in human behavior, this study denotes trust and communication in the interaction behavior of industry–university–research cooperation as cooperative trust and cooperative communication, respectively.

### Interactive behavior and cooperative innovation performance of industry–university–research cooperation

Cooperative trust means that, in order to achieve the expected cooperative goals, enterprises believe universities and research institutes are capable and willing to fulfill their commitments as expected, and are willing to take the risks associated with opportunistic behaviors and others. That is, it is believed that universities and research institutes will provide true and useful explicit and implicit knowledge as required, and will not take advantage of a enterprises’ weaknesses for profits (Li et al., 2015). According to transaction cost theory, although industry–university–research cooperation has the advantages of reducing R&D uncertainty, shortening R&D time costs, and optimizing resource allocation to reduce transaction costs (Kafouros et al., 2015; Wang et al., 2021; Cui and Li, 2022), there are also certain problems of rising transaction costs in industry–university–research cooperation, such as the cost of finding partners, the cost of cooperative communication, the cost of digesting the asymmetry between theoretical research and actual needs, and the potential risk cost of knowledge leakage (Wang and Hu, 2022). The increase in the above costs will reduce some enterprises’ willingness to cooperate, or reduce the input of cooperation. The reason for this is that enterprises do not have enough trust in universities’ technical ability and emotion. This virtually increases the cost of evaluation. Li pointed out that the trust mechanism is a prerequisite and foundation for innovation in industry–university–research cooperation, and it is a soft governance mechanism that can make up for the shortcomings of the formal management mechanisms (Li et al., 2015). Industry–university–research cooperative trust not only affects the psychological process of cooperative innovation subjects, reduces the expectation of the risks faced by cooperative innovation, and improves the willingness of participants to cooperate in innovation and the level of participation in cooperative innovation, but also directly and indirectly affects people’s attitudes, expectations, behaviors, and performance (Dirks, 1999; Dirks and Ferrin, 2001; Xu and Gao, 2006). Secondly, the mutuality between the subjects of industry–university–research cooperation will increase information sharing, teamwork, and other behaviors, improve information exchange, and thus improve performance (Zhang et al., 2020). This influence can be explained from two perspectives. From the perspective of knowledge base, the cooperative trust between subjects can effectively promote knowledge sharing, knowledge transfer, and learning innovation among members (Li and Liu, 2014; De Zubielqui et al., 2019; Wang et al., 2021). From the perspective of knowledge transfer, industry–university–research cooperative trust can reduce the uncertainty of knowledge exchange, inactivity, increase the frequency and success rate of knowledge transfer, improve the outcome of knowledge exchange, and thus improve cooperative performance (Hardwick et al., 2013). Takagi, D had verified that the spatial Durbin model showed that spatially weighted neighborhood trust was positively associated with behaviors (Takagi and Shimada, 2019). On the basis of the above analysis, the following hypothesis is proposed:

*Hypothesis 1* *(H1)*. Cooperative trust in industry–university–research cooperation is positively correlated with cooperative innovation performance.

Industry–university–research cooperative communication refers to the communication process of obtaining, transferring, and understanding information between the members of the cooperation subjects in order to accomplish the goal of cooperation, which can be divided into formal communication, such as seminar meetings, on-site guidance, and appropriate training and informal language communication according to the organizational form of cooperation communication. The essential goal of industry–university–research cooperation is to realize the knowledge transfer. Knowledge transfer is actually a communication process between the knowledge transmitter and the knowledge receiver (Li et al., 2022). The effect of cooperative communication on cooperative innovation performance mainly depends on three mechanisms: Firstly, cooperative communication reduces information asymmetry and cooperation risks; secondly, cooperative communication promotes the flow and transfer of knowledge; thirdly, cooperative communication optimizes resource the allocation of resource. In industry–university–research cooperation, there are asymmetries between technology supply and technology demand, and between theoretical research and practical needs. In addition, there are information asymmetries such as market changes and untimely changes in the technical parameters in the actual cooperation process, which increase the cooperation costs and cooperation risks of enterprises. Practice has proved that intentional communication can maintain the consistency of psychological state between subjects and form cooperative communication (Vasil et al., 2020), and strengthening cooperative communication can greatly reduce information asymmetry and cooperative cultural conflict (Kopelman et al., 2016). In addition, the essence of the goal of industry–university–research cooperation is to realize knowledge transfer (Li et al., 2022). Knowledge transfer is defined as a communication process between the knowledge transmitter and the knowledge receiver (Li et al., 2022). Cooperative communication between industry, university, and research institutes can broaden the breadth and depth of knowledge transfer channels, reduce the barriers to knowledge transfer and realize knowledge transfer more efficiently (Dong et al., 2005). However, universities and research institutes are in the “knowledge highland,” while enterprises are in the “knowledge lowland,” and there is a certain “gap” in knowledge. Through the cooperation of industry, university, and research, the knowledge “gap” can be filled and resources can be realized. However, industry–university–research cooperation is formed *via* a static relationship (Li and Liu, 2013). Resource complementation must be realized through communication; otherwise, cooperation is only a formality. Finally, as verified using game theory, industry–university–research cooperation can achieve better resource allocation (Huyck et al., 1990; Yang et al., 2008). Huyck et al. (1990) suggested that the game between industry–university–research cooperation is consistent with the prisoner’s dilemma, which can be achieved by repeatedly playing the same coordination game in order to increase the probability of Pareto-optimal Nash equilibrium. Suppose that, in the process of industry–university–research cooperation, the university dominant strategy benefits EU1, and the enterprise dominant strategy benefits EC1. Thus in the absence of cooperative communication and incomplete information, the total benefit is E1 = EU1 + EC1; however judging from cooperative economics, both parties cooperate and the benefit after information sharing is E, as the benefit of optimal strategy is E > E1, so the efficiency of cooperative communication is higher. But, the quality of communication will also affect performance (Gonzalez-Roma and Hernandez, 2014; Valls et al., 2016).On the basis of the above analysis, the following hypothesis is proposed:

*Hypothesis 2* *(H2)*. Cooperative communication in industry–university–research cooperation is positively correlated with cooperative innovation performance.

### Interactive behavior of industry–university–research and knowledge absorptive capacity

Cohen and other scholars first proposed the concept of knowledge absorptive capacity. They believed that knowledge absorptive capacity includes the ability of enterprises to identify and acquire external knowledge, digest it and apply it within the enterprise (Cohen and Levinthal, 1990). On the basis of Cohen’s definition, many other scholars divide knowledge absorptive capacity into four segments: knowledge acquisition, knowledge assimilation, knowledge transformation, and knowledge exploitation (Schweisfurth and Raasch, 2018; Meng et al., 2021). Among them, knowledge acquisition and knowledge assimilation are defined as potential absorptive capacity, and knowledge transformation and knowledge exploitation are defined as real absorptive capacity. In the industry–university–research cooperation relationship, parties having a high degree of trust in each other promote enterprises to establish positive behavioral expectations, making them more willing to take risks and actively share information (Norman, 2004). This helps to improve their own knowledge absorptive capacity. First of all, cooperative trust in industry–university–research cooperation means both parties do not need to spend more time and effort in establishing monitoring mechanisms and bargaining, saving time costs that can be spent on knowledge acquisition. Motivated by cooperative trust, universities and research institutes are more willing to share knowledge and ideas to promote knowledge acquisition, and enterprises are more willing to share valuable and trustworthy information, simplifying the process of knowledge acquisition, which, in turn, helps to improve the enterprise’ knowledge acquisition capacity (Li et al., 2015). Secondly, cooperative trust can increase the frequency of communication and interaction, increasing communication flow. It enables both sides to have more opportunities to discuss and communicate in depth about the difficulties existing in the knowledge interaction, and transfer and express the knowledge in an easily understandable form. This is conducive cooperative enterprises improving their knowledge assimilation capacity and knowledge absorption capacity the cooperative enterprises (He et al., 2021). However, Davenport and Prusak argue that the idea of “not invented here” hinders the application of externally acquired knowledge. Therefore, organizational trust is needed when using external knowledge. They also suggested that organizational trust would be more conducive to knowledge absorption and exploitation (Davenport and Prusak, 2000). Therefore, cooperative trust in the industry–university–research partnership is a non-institutional arrangement through which enterprises encourage the use of external knowledge, which helps to improve the enterprises’ knowledge absorptive capacity. On the basis of the above analysis, the following hypothesis is proposed:

*Hypothesis 3* *(H3)*. Cooperation trust in industry–university–research cooperation is positively correlated with knowledge absorptive capacity.

Cooperative communication in university–industry–research is the basis for knowledge acquisition, knowledge assimilation, knowledge transformation, and knowledge exploitation in university–industry–research cooperation (Xie et al., 2018). Industry–university–research cooperation is also a platform for building knowledge exchange and learning. Universities and research institutes can be seen as knowledge exporters and enterprises as knowledge receivers. Thus, in the process of exporting and receiving, continuous explanation and communication can enable enterprises to understand and master knowledge better and faster. In turn, it will promote their knowledge absorptive capacity. From the perspective of knowledge learning theory, cooperative communication promotes the realization of knowledge absorptive capacity mainly through the following means. Firstly, cooperative communication simplifies the process and practice of knowledge screening, which can save knowledge acquisition costs; secondly, cooperative communication can explain and illustrate explicit knowledge, helping enterprises to understand, digest, and absorb knowledge; thirdly, cooperative communication can also produce knowledge spillover effects, helping enterprise employees to establish the correct learning concept, learning methods, and approaches, and improve their knowledge utilization capacity. The above views have been supported by a number of scholars. Xie et al. (2018) and others use exploratory case analyses to demonstrate that the establishment of communication and exchange mechanism is inseparable from any stage of industry–university–research cooperation, and through communication and exchange, the ability of enterprises to acquire, digest, integrate and, utilize knowledge is improved. However, in addition to sharing explicit knowledge between the subjects of industry–university–research cooperation, there is also a larger sharing of tacit knowledge, which is not easily encoded, and the knowledge sender and receiver need to share tacit knowledge through close interactive communication (Qiu and Haugland, 2019), which improves the knowledge reception ability of members. Finally, industry–university–research cooperation communication reduces information asymmetry and cooperation risks, making cooperation members more willing to cooperate and take the initiative to improve their knowledge absorptive capacity. On the basis of the above analysis, the following hypothesis is proposed:

*Hypothesis 4* *(H4)*. Cooperative communication in industry–university–research cooperation is positively correlated with knowledge absorptive capacity.

### The mediating effect of knowledge absorptive capacity

The relationship between knowledge absorptive capacity and innovation performance is very well established and it is believed that knowledge absorptive capacity has an important positive contribution to innovation performance (Casanueva et al., 2013; Li and Zhang, 2020; Li and Zhu, 2021). Knowledge absorptive capacity can promote knowledge transfer, which, in turn, has an important positive effect on innovation performance (Casanueva et al., 2013; Li and Zhang, 2020; Li and Zhu, 2021), That is, as the knowledge absorptive capacity of enterprises increases, the innovation performance also increases (Zhou, 2022). The effect is manifested in several ways. On the one hand, the stronger the potential knowledge absorptive capacity, the more effective a enterprise is at identifying the new knowledge needed and at absorbing and utilizing it (Braojos et al., 2020). In contrast, actual absorptive capacity as an exploitative learning processes enables the translation and exploitation of absorbed knowledge (Braojos et al., 2020; Miroshnychenko et al., 2021). On the other hand, enterprises with a strong knowledge absorptive capacity have good internal knowledge digestion and reorganization mechanisms, which can increase the speed and frequency of innovation (Meng et al., 2021). Moreover, enterprises with strong knowledge absorptive capacity are better able to benefit from the corresponding knowledge transfer process (Zhao et al., 2019). In studying the relationship between knowledge absorptive capacity and enterprise innovation, Gao also found that knowledge absorptive capacity has a significant positive impact on enterprise innovation performance and that enterprises exhibit innovation inertia due to knowledge absorption (Gao and Zhang, 2019).

The above analysis focuses on the process through which knowledge absorptive capacity can promote knowledge acquisition, knowledge assimilation, knowledge transformation, and knowledge exploitation. The interactive behavior of the cooperative subjects of industry–university–research cooperation increases the sharing and exchange of knowledge and provides channels and possibilities for knowledge flow. However, the knowledge acquired by enterprises from the outside cannot be directly transformed into the enterprise’s innovation performance. External knowledge must be absorbed and digested before it can be transformed into actual output (Cohen and Levinthal, 1990). Knowledge absorptive capability, as a dynamic capability, not only helps enterprises to identify knowledge, but also promotes the knowledge absorption, knowledge assimilation, and knowledge transformation. It plays the role of a “connector” (Zhang et al., 2022). It has been shown that knowledge absorptive capacity plays an important mediating role in using external knowledge to improve the enterprise’s innovation performance (Ye and Chen, 2022; Zhang et al., 2022). The above analysis table shows that the interactive behavior of industry–university–research cooperation subjects helps to improve enterprise’s knowledge absorptive capacity, and knowledge absorptive capacity promotes enterprise’s innovation performance. Therefore, the interactive behavior of industry–university–research cooperation can promote cooperative innovation performance by influencing knowledge absorptive capacity. On the basis of the above analysis, the following hypothesis is proposed:

*Hypothesis 5* *(H5)*. Knowledge absorptive capacity mediates the relationship between interactive behavior and cooperative innovation performance.

**Figure 1:**
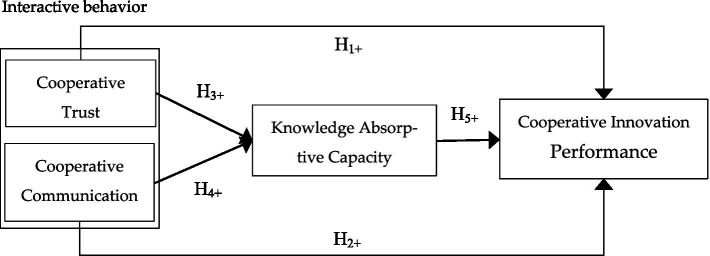
Theoretical model. [+] plus sign indicates positive relationships, and [→] solid line indicates direct relationships.

The above analysis led to the theoretical research model, as shown in Figure 1.

## Methodology

### Measurement of variables

All the variables covered in this study were obtained by means of a questionnaire. The items for the constructs were assessed using a five-point Likert scale, ranging from 1 = “strongly disagree” to 5 = “strongly agree.”

#### Cooperative trust (CT)

Li and Liu (2013) divided the trust in industry–university–research cooperation into three dimensions: computational trust, cognitive trust, and identity trust, and developed 12 measurement indicators based on previous research results. Unlike the study by Li, this study mainly focuses on technical trust and emotional trust. Technical trust refers to the belief that universities and research institutes are capable of overcoming technical problems. Emotional trust refers to the belief that universities and research institutes are willing to help solve technical problems and are sincere in their cooperation. On the basis of the above concepts, combined with the need for cooperative trust, and taking into account the studies of Nguyen, Luu and Trong, Mc Allister, and others, cooperative trust was measured using two constructs and six items (e.g., “enterprises believe in the ability of cooperative universities and their preparation for work” and “professional knowledge of cooperative universities can help enterprises solve practical problems”) (Trong Hai et al., 2014).

#### Cooperative communication (CC)

The Cooperative Communication Scale was referenced from Fan’s study (Fan et al., 2010). Combined with the research content of this paper, four professional doctors were invited to discuss and initially determine the items. After a small-scale preinvestigation, five items were identified (e.g., “the partners are able to have timely discussions on issues in the study”).

Knowledge Absorptive Capacity (KAC). This paper continues to classify knowledge absorptive capacity into two constructs according to four aspects of knowledge acquisition, assimilation, transformation, and exploitation. They are denoted potential knowledge absorptive capacity and real knowledge absorptive capacity. On the basis of the studies of various scholars (Li and Zhang, 2020; Linh Nguyen et al., 2022), the research team discussed the interviews and summaries from the research leaders of four enterprises in Zhejiang. After the prestudy, two constructs and 11 items were finally identified (e.g., “The enterprise is able to quickly determine the value and usefulness of new external knowledge to existing knowledge” and “The enterprise has the ability to organize and use new knowledge that has been acquired”).

#### Cooperative innovation performance (CIP)

Following the work of foreign scholars (Wu et al., 2017; Sun et al., 2019), combined with the study results of Fan et al. (2010), and considering the connotation of industry–university cooperative innovation performance in this paper, we finally decided to measure cooperative innovation performance using two constructs and six items. The two constructs are learning growth performance and innovation task performance (e.g., “enterprises can master certain knowledge about R & D innovation through industry–university–research cooperation” and “The project achieved the expected technological innovation requirements”).

#### Control variables

Referring to the studies of Chen et al. (2020), Li et al. (2020), Zhang et al. (2022) and other scholars, enterprise age (Age), enterprise size (Size), and industry–university–research experience (Exp) were selected as control variables (Chen et al., 2020; Li and Zhang, 2020; Zhang and Chen, 2022). This paper argues that enterprises that have been in business for a long time are more sensitive to market information, which facilitates the accumulation of innovation resources and a priori knowledge, and therefore enhances their innovation capacity. Larger enterprises have the strength and capital to carry out corporate management and engage in product innovation activities, but there is also a certain degree of rigidity in enterprises of this size, which is not conducive to innovation. Enterprises’ willingness to innovate and innovative behavior are also closely related to their past experience of cooperation. The value of 1 was assigned to denote experience of industry–university–research, while 0 was assigned to denote the absence of such experience.

### Survey procedures and sample selection

For the survey, we chose Zhejiang Province of China for the following reasons: (1) China attaches great importance to industry–university–research cooperation, and has issued a number reports to this end; (2) In 2020, the number of Chinese R&D institutions was 6,682; (3) the industrial R&D expenditure of above-scale industries in Zhejiang Province reached CNY13,988,988 million, ranking third in China; (4) new product development expenditure for industrial enterprises above the scale reached CNY1,762,995,000, ranking third in China; (5) Zhejiang Province has 109 higher education institutions, and 105,887 scientific and technological personnel, and cooperative supply resources are abundant; (6) in 2021, there were 49,876 research and development projects in the field of natural science in higher education institutions in Zhejiang Province, which is a solid foundation for cooperation; and (7) The authors all work in Zhejiang universities and are in contact with the local Science Technology Department, which was highly convenient.

In order to gain a deeper understanding of the relationship between the interactive behavior of industry–university–research cooperation and the cooperative innovation performance, this study selected three enterprises that we were in contact with for interviews and the case rooting analysis before the formal questionnaire was administered. Then, the questionnaire was designed and iterated after considering the relevant literature, the opinions of experts, and the opinions of the responsible individuals in the enterprises concerned. On the basis of a small-scale preinvestigation of 50 participants from enterprises in the innovation and entrepreneurship training course hosted by the Zhejiang Education Development Centre in 2021, the scale was revised and refined to produce the final questionnaire.

The formal survey was mainly conducted with the help of the platform of the Zhejiang-China Development Research Institute. Through various local cooperation projects, especially with the local Science Technology Department, we requested cooperation of relevant individuals from this department, to conduct the research. The collection procedures were as follows: Firstly, we requested the support of the leaders of the Science Technology Department, and explained the purpose of the survey, our methodology, and things of note to these individuals. Secondly, we sent the electronic questionnaire created by the team to the leaders. Thirdly, the leaders gave us the contact details of the leaders. We contacted the individuals and explained the purpose of the survey and the requirements for filling it out. Fourthly, the questionnaire was sent to the leaders, who contacted the research leaders regarding filling it out. This survey took nearly 2 months in total. A total of 400 questionnaires were distributed and 325 valid questionnaires were returned, with an effective rate of 81.25%. The sample was distributed in various regions of Zhejiang Province. We consider the sample valid. The statistics of the surveyed sample enterprises are shown in Table 1.

**Table 1:** Characteristics of the sample.

Statistical variables	Classification	Percentage	Projects	Statistical variables	Percentage
Enterprise age	Less than 3 years	7.4	Industry	Food/cigarettes	2.8
	3–5 years	25.2		Electronic	2.8
	5–10 years	27.1		Metal/mechanical engineering	10.8
	10–20 years	28		Textile/clothing	5.8
	More than 20 years	12.3		Electronic equipment	17.2
Enterprise size	Under 100 people	24.6		Building materials	8.3
	101–200 people	19.1		Transport equipment	4.3
	201 ~ 500 people	48.6		Software	10.5
	501 ~ 1,000 people	5.2		Pharmaceutical/medical	1.2
	Over 1,000 people	2.5		Rubber/plastic	10.5
Number of researchers	10 and under	42.8		Petroleum/chemicals	6.8
	11–20 people	31.7		Printing/Publishing	2.8
	20–40 people	23.7		Jewelry	5.2
	41–80 people	1.2		Timber	7.1
	81+ people	0.6		Other	4.0

(*n* = 325). Source: Compiled by the author.

## Data analysis and results

### Reliability, validity, and common method deviation test

As for the common method deviation, on the one hand, an anonymous questionnaire and other methods were used to control in advance. On the other hand, the Harman one-way test was used to test. The results showed that the cumulative variance contribution rate of the first factor was 32.801 < 40%, indicating that there was no homologous deviation in the study.

In this paper, the orthogonal maximum variance method of the exploratory factor analysis was used to conduct the factor composition analysis and then to explore the structural validity of the questionnaire. The internal consistency coefficient Cronbach’s Alpha, Kaiser–Meyer–Olkin (KMO) sample measure, and Bartlett’s sphere test were used to test the reliability of the questionnaire. The internal consistency coefficient Cronbach’s Alpha analysis was conducted using the SPSS 23.0 software. As shown in Table 2, all variables were tested.

**Table 2:** Reliability and validity tests.

Variables	# of items	Reliabilities	Validity
Cooperative trust	6	0.769	0.762
Cooperative communication	5	0.837	0.853
Knowledge absorptive capacity	11	0.891	0.820
Cooperative innovation performance	6	0.830	0.668

### Confirmatory factor analysis

In this study, we conducted the confirmatory factor analysis using Amos 23.0 to test the discriminant validity of cooperative trust, cooperative communication, knowledge absorptive capacity, and cooperative innovation performance. As shown in Table 3, each fitting index of the four-factor model met the critical standard, and the fitting effect of the model was significantly better than that of other models. The analysis results show that the discrimination validity among the four variables was high, and the fitting effect of the four-factor model was good.

**Table 3:** Results of confirmatory factor analysis.

Model description	Model inclusion factors	x_2_/df	RMAEA	IFI	CFI	NFI
Four-factor model	CT, CC, KAC, CIP	1.717	0.047	0.959	0.959	0.908
Three-factor model	CT + CC, KAC, CIP	2.647	0.071	0.904	0.903	0.854
Three-factor model	CT + KAC, CC, CIP	4.102	0.098	0.816	0.815	0.771
Three-factor model	CT, CC + KAC, CIP	5.406	0.117	0.737	0.735	0.696
Two-factor model	CT + CC + KAC, CIP	4.476	0.104	0.794	0.792	0.750
One-factor model	CT + CC + KAC + CIP	4.666	0.106	0.785	0.783	0.742

“+” represents the combination of the two factors before and after into one variable, *N* = 325. Source: compiled by the authors.

### Correlation analysis and inspection

The results of the correlation analysis showed the correlation coefficient, mean, and variance of each variable. As shown in Table 4, cooperative trust had a significant positive correlation with knowledge absorptive capacity (*r* = 0.465, *p* < 0.01), and cooperative innovation performance (*r* = 0.369, *p* < 0.01). Cooperative communication was positively related to knowledge absorptive capacity (*r* = 0.394, *p* < 0.050), and cooperative innovation performance (*r* = 0.433, *p* < 0.01). Finally, there was a significant positive correlation between knowledge absorptive capacity and cooperative innovation performance (*r* = 0.545, *p* < 0.01).

**Table 4:** Correlation analysis results of research variables.

Variables	*M*	SD	Age	Size	Exp	CT	CC	KAC	CIP
Age	3.1262	1.144	1						
Size	2.3662	1.027	0.032	1					
Exp	0.6492	0.478	−0.049	−0.065	1				
CT	3.363	0.622	0.069	−0.098	0.050				
CC	3.104	0.738	−0.009	−0.125^*^	−0.007	0.682^**^			
KAC	3.603	0.632	0.009	−0.053	−0.025	0.465^**^	0.394^**^		
CIP	3.099	0.817	−0.067	−0.012	−0.028	0.369^**^	0.433^**^	0.545^**^	1

^**^denotes significantly correlated at the 0.01 level (two-sided); ^*^denotes significantly correlated at the 0.05 level (two-sided). Numbers in brackets are internal consistency coefficients. Source: Compiled by the author.

### Hypothesis tests

Table 5 shows the results of the hypothesis test. Firstly, according to the results of model 1 and model 2, after controlling for age, size, and experience, the regression coefficient of cooperative trust was 0.379 (*β* = 0.379, *p* < 0.050) and the regression coefficient of cooperative communication was 0.120 (*β* = 0.120, *p* < 0.050). That is, if cooperative trust and cooperative communication are high, the knowledge absorptive capacity is better. Thus, the hypotheses H_3_ and H_4_ are verified. Secondly, according to model 3 and model 4, cooperative trust and cooperative communication (*β* = 0.200, *p* < 0.050; *β* = 0.370, *p* < 0.001) have a significant positive effect on cooperative innovation performance. Thus, the hypotheses H1 and H2 are verified. Using model 4 and model 6, cooperative trust, cooperative communication, and knowledge absorptive capacity were simultaneously entered into the regression. The results show that cooperative communication and knowledge absorptive capacity (*β* = 0.301, *p* < 0.001; *β* = 0.578, *p* < 0.001) have a significant positive effect on cooperative innovation performance. Whereas, cooperative trust (*β* = −0.019, *p* > 0.05) has no significant positive effect on cooperative innovation performance. Therefore, it can be concluded that knowledge absorptive capacity plays a partial intermediary role between interactive behavior and cooperative innovation performance. Thus, hypothesis H5 is verified.

**Table 5:** Results of regression analysis.

Variables	KAC		CIP			
	Model 1	Model 2	Model 3	Model 4	Model 5	Model 6
Age	0.005 (0.031)	−0.009 (0.027)	−0.049 (0.040)	−0.056 (0.036)	−0.053 (0.033)	−0.050 (0.032)
Size	−0.034 (0.034)	−0.001 (0.031)	−0.009 (0.044)	0.036 (0.040)	0.015 (0.037)	0.036 (0.036)
Exp	−0.037 (0.074)	−0.058 (0.065)	−0.055 (0.095)	−0.059 (0.086)	−0.029 (0.080)	−0.026 (0.077)
CT		0.379^**^ (0.069)		0.200^**^ (0.090)		−0.019 (0.085)
CC		0.120^**^ (0.058)		0.370^***^ (0.076)		0.301^***^ (0.069)
KAC					0.706^***^ (0.060)	0.578^***^ (0.066)
R^2^	0.004	0.229	0.006	0.207	0.303	0.361
Adjusted R^2^	−0.006	0.217	−0.004	0.194	0.294	0.349
Amount of change in F	0.400	46.631	0.610	40.384	136.303	76.605
Maximum VIF	1.006	1.901	1.006	1.901	1.008	2.081

^**^p < 0.01, ^***^p < 0.001 (two tails). Source: Compiled by the author.

In this study, the bootstrap method was used to verify the mediation. The data show that the indirect effect of cooperative trust on cooperative innovation performance through knowledge absorptive capacity was 0.2902, with a BootLLCI of 0.2098 and a BootULCI of 0.3812, and the indirect effect of cooperative communication on cooperative innovation performance through knowledge absorptive capacity was 0. 1933, with a BootLLCI of 0.1293 and a BootULCI of 0.2666. The range does not include 0. Therefore, knowledge absorptive capacity plays a significant mediating role in the relationship between interactive behavior and cooperative innovation performance.

### Heterogeneity analysis

Firstly, in order to investigate the impact of the interactive behavior of industry–university–research cooperation on the cooperative innovation performance of enterprises of different scales, we took the number of cooperative enterprises as a proxy variable for the size of the enterprises, and divided the enterprises with 200 persons or less in the whole sample into “enterprise size_low” and those with more than 200 persons are divided into “enterprise size_high” for regression. The results are shown in Table 6.

**Table 6:** Heterogeneity analysis of the enterprise scale.

Variables	Enterprise size_low				Enterprise size_high			
	KAC		CIP		KAC		CIP	
	Model 1	Model 3	Model 2	Model 4	Model 5	Model 6	Model 7	Model 8
Age	0.017 (0.038)	−0.015 (0.047)	−0.015 (0.053)	−0.025 (0.047)	0.001 (0.041)	−0.099^*^ (0.050)	−0.057 (0.052)	−0.058 (0.047)
Exp	−0.170^*^ (0.102)	0.078 (0.129)	−0.021 (0.141)	0.083 (0.128)	0.023 (0.083)	−0.130 (0.101)	−0.101 (0.104)	−0.113 (0.095)
CT	0.256^**^ (0.107)		0.126 (0.147)	−0.030 (0.135)	0.472^***^ (0.088)		0.254^**^ (0.110)	0.009 (0.109)
CC	0.070 (0.094)		0.252^*^ (0.130)	0.210^**^ (0.117)	0.154^**^ (0.072)		0.448^***^ (0.090)	0.368^***^ (0.083)
KAC		0.663^***^ (0.099)		0.612^***^ (0.104)		0.734^***^ (0.076)		0.519^***^ (0.087)
*R* ^2^	0.127	0.236	0.080	0.261	0.353	0.372	0.352	0.464
Adjusted *R*^2^	0.103	0.220	0.055	0.235	0.338	0.361	0.337	0.448
Amount of change in *F*	5.246	14.943	3.147	34.834	23.368	33.981	23.193	35.652
Maximum VIF	1.934	1.087	1.934	2.012	1.928	1.019	1.928	2.221

^*^p < 0.05, ^**^p < 0.01, ^***^p < 0.001 (two tails). Source: Compiled by the author.

By comparing the regression coefficients in the table, we found that knowledge absorptive capacity moderated the effect of cooperative innovation performance in both groups. In the “enterprise size_high” group, knowledge absorptive capacity played a complete intermediary role between cooperative trust and cooperative innovation performance, and a partial intermediary role between cooperative and cooperative innovation performance. In the “enterprise size_low” group, knowledge absorptive capacity played a partial intermediary role between cooperative communication and cooperative innovation performance. In this group, the relationship between trust and innovation performance was not tested (Table 6).

Secondly, according to the age index from small to large, this study divides the enterprises into two groups. Enterprises aged under 10 years (and including 10 years) are named “low-group” and enterprises aged over 10 years are named “high-group.” By comparing the regression coefficients of the two groups, we found that knowledge absorptive capacity played a complete intermediary role between cooperative trust and cooperative innovation performance in the two groups, and a partial intermediary role between cooperative communication and cooperative innovation performance in the two groups. However, the relationship between cooperative trust and cooperative innovation performance was not verified in the “high-group” (Table 7).

**Table 7:** Heterogeneity analysis of enterprise age.

Variables	Low-Group				High-Group			
	KAC		CIP		KAC		CIP	
	Model 1	Model 2	Model 3	Model 4	Model 5	Model 6	Model 7	Model 8
Size	0.043 (0.045)	0.013 (0.055)	0.048 (0.059)	0.021 (0.053)	−0.056 (0.045)	−0.021 (0.054)	−0.011 (0.059)	0.019 (0.054)
Exp	0.002 (0.085)	−0.078 (0.104)	−0.064 (0.113)	−0.066 (0.101)	−0.147 (0.102)	0.016 (0.127)	−0.064 (0.134)	0.016 (0.123)
CT	0.348^***^ (0.086)		0.216^*^ (0.114)	0.005 (0.106)	0.355^**^ (0.120)		0.137 (0.157)	−0.058 (0.148)
CC	0.167^**^ (0.076)		0.376^***^ (0.101)	0.274^**^ (0.091)	0.058 (0.090)		0.357^**^ (0.117)	0.325^**^ (0.107)
KAC		0.758^***^ (0.076)		0.608^***^ (0.086)		0.622^***^ (0.103)		0.547^***^ (0.106)
*R* ^2^	0.277	0.247	0.236	0.391	0.170	0.237	0.154	0.302
Adjusted *R*^2^	0.261	0.337	0.220	0.381	0.143	0.219	0.127	0.274
Amount of change in *F*	18.065	33.710	14.627	50.154	6.440	13.183	5.722	26.614
Maximum VIF	2.090	1.007	1.934	2.271	1.674	1.058	1.674	1.790

^*^p < 0.05, ^**^p < 0.01, ^***^p < 0.001 (two tails). Source: Compiled by the author.

Thirdly, this study investigated whether the previous experience of industry–university–research cooperation affects the cooperative innovation performance. In this study, the enterprises were divided into two types of samples, the “inexperienced group” and the “experienced group.” As shown in Table 8, enterprises with previous experience in cooperation considered cooperative trust to be important for knowledge absorption capacity and cooperative innovation performance. The lack of cooperative trust made them more concerned about “hitchhiking” on research and the risk of intellectual property leakage. For enterprises with no prior experience, cooperative trust had a relatively small impact on cooperative innovation performance, while cooperative communication had a relatively large impact on cooperative innovation performance.

**Table 8:** Heterogeneity analysis of enterprise cooperation experience.

Variables	Inexperienced Group				Experienced Group			
	KAC		CIP		KAC		CIP	
	Model 1	Model 2	Model 3	Model 4	Model 5	Model 6	Model 7	Model 8
Age	0.016 (0.041)	−0.073 (0.050)	−0.048 (0.052)	−0.057 (0.048)	−0.023 (0.037)	−0.044 (0.045)	−0.059 (0.049)	−0.045 (0.044)
Size	−0.045 (0.046)	0.074 (0.058)	0.063 (0.060)	0.088 (0.055)	0.029 (0.041)	−0.022 (0.049)	0.017 (0.054)	0.000 (0.048)
CT	0.399^**^ (0.126)		0.070^*^ (0.162)	−0.148 (0.154)	0.349^**^ (0.083)		0.237^**^ (0.110)	0.029 (0.103)
CC	0.193^*^ (0.108)		0.554^***^ (0.139)	0.448^**^ (0.129)	0.090 (0.069)		0.302^***^ (0.092)	0.247^**^ (0.083)
KAC		0.726^***^ (0.098)		0.546^***^ (0.113)		0.706^***^ (0.103)		0.598^***^ (0.083)
*R* ^2^	0.348	0.336	0.292	0.419	0.178	0.291	0.171	0.340
Adjusted *R*^2^	0.324	0.317	0.266	0.392	0.162	0.280	0.155	0.324
Amount of change in F	14.518	18.515	11.255	23.556	11.172	28.285	10.647	52.360
Maximum VIF	2.487	1.007	2.452	2.523	1.716	1.002	1.716	1.851

^*^p < 0.05, ^**^p < 0.01, ^***^p < 0.001 (two tails). Source: Compiled by the author.

**Figure 2:**
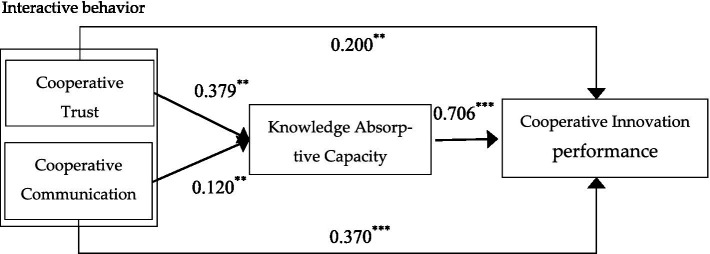
The full model. ^**^p < 0.01. ^***^p < 0.001.

According to the above analysis, the hypothesis is basically supported, as shown in Figure 2.

## Discussion

The study examines the relationship between interactive behavior (cooperative trust and cooperative communication), knowledge absorptive capacity, and cooperative innovation performance based on the perspective of knowledge learning theory. Using enterprises from Zhejiang Province as research subjects, the study reveals that interactive behaviors promote cooperative innovation performance, and that knowledge absorptive capacity plays a mediating role.

Firstly, the interactive behavior of industry–university–research cooperation has a positive impact on cooperative innovation performance. The results of this study are consistent with those of existing studies (Li and Liu, 2013). On basis of transaction cost theory, a higher the degree of cooperative trust in the cooperation of industry–university–research, reduces the explicit and implicit cost assessment from a psychological perspective. In turn, it can increase their willingness to cooperate and the initiative of knowledge sharing (Dirks and Ferrin (2001). On the other hand, in industry–university–research cooperation, cooperative trust not only strengthens mutual willingness to share knowledge and accelerate knowledge flow, long-term cooperative trust can even form a culture of cooperation, reducing knowledge protection and opportunistic behavior and promoting effective knowledge flow (Wu et al., 2022). It has been demonstrated that effective knowledge transfer must be achieved through communication. Effective cooperative communication helps to increase the breadth and depth of knowledge transfer channels and ensures smooth knowledge transfer (Dong et al., 2005). The results of this study also support this point of view.

Secondly, interactive behavior promotes the improvement of knowledge absorptive capacity. The trust mechanism is an important part of cooperation. Trust can increase the initiative of knowledge sharing and improve exploratory and exploitative learning capabilities (Wang and Hu, 2022). Cooperative communication can lead to more knowledge spillover effects. This all offers the potential to increase the knowledge absorptive capacity of enterprises (Pinjani and Palvia, 2013; Zhao et al., 2019; Braojos et al., 2020). The findings of the study also further validate the theoretical hypothesis.

Thirdly, knowledge absorptive capacity plays a partially mediating role between the interactive behavior and cooperative innovation performance. It was shown that knowledge absorptive capacity partially mediated the relationship between cooperative communication and cooperative innovation performance and completely mediated the relationship between cooperative trust on cooperative innovation performance. Ye and Chen (2022) also verified the existence of a partially mediating role of knowledge absorptive capacity and innovation performance (Ye and Chen, 2022). This study demonstrates that knowledge absorptive capacity plays a completely and partially mediating role between interactive behavior and cooperative innovation performance. A possible explanation for this is that if the industry–university–research cooperation remains on a trust basis and there is no activity, it is unlikely that performance is generated, i.e., there is no action, although there is the possibility of knowledge sharing. Therefore, knowledge absorptive capacity is needed to externalize the flow of knowledge, as cooperative communication itself is a dynamic communication. As long as it is not a “closed” state, employees will always consider transforming knowledge and improving performance. Therefore, it partially mediates cooperative communication and cooperative innovation performance (Valentim et al., 2016).

Fourthly, the heterogeneity analysis also produced interesting conclusions. The mediating role of knowledge absorptive capacity is significant in larger enterprises, but in the smaller enterprises, the mediating role is only significant in the relationship between cooperative communication and cooperative innovation performance. It can be inferred that the larger enterprises pay more attention to the growth of their own R&D capacity, and pay more attention to the improvement of their knowledge absorptive capacity, especially their potential knowledge absorption capacity. Their sense of “self-improvement” is more prominent. They pay particular attention to exchange, learning, and growth. This finding further supports the research of many scholars who postulate that enterprise size is positively related to learning capacity (Cao et al., 2013; Lu and Ye, 2017). For relatively young enterprises, cooperative communication and cooperative trust promote knowledge absorptive capacity, whereas cooperative trust in older enterprises does not promote knowledge absorptive capacity. This may be due to the fact that older enterprises develop a sense of territoriality, cognitive entrenchment, or even a “covering effect,” resulting in distrust or even rejection of foreign technology (Zhao and Ma, 2021). Young enterprises are not “leaders,” so they must strengthen their own viability. They must strive to improve their knowledge absorption capacity. In addition, enterprises with previous experience in industry–university–research cooperation are more likely to recognize cooperative trust. A possible explanation for this is that university–industry–research cooperation increases transaction costs, which is of concern to enterprises with previous experience, but enterprises without previous cooperation experience have a low perception of the risk of a lack of cooperative trust. This also suggests that any entities engaged in industry–university–research cooperation must weigh the risks and benefits, and must avoid conflicts of interest; otherwise, it will affect the subsequent industry–university–research cooperative behavior (Norman, 2004; Xu and Gao, 2006).

## Implications, conclusion, and final remarks

### Theoretical implications

Firstly, this study breaks the macroperspective of existing research regarding the effects of cooperative innovation performance, and extends to the exploration of the microperspective. The study proposes the interactive behavior of industry–university–research from a micro perspective, and defines and analyzes the dimensions of the interactive behavior. It echoes the research of certain other scholars, such as Li and Ye (2011), Li and Liu (2013), and Liu (2013), and also broadens our perspectives regarding how to improve the cooperative innovation performance. Secondly, this study formally delineates interactive behavior dimensions. It classifies them into cooperative trust and cooperative communication, and verifies their effects on cooperative innovation performance, providing a theoretical analysis tool for subsequent research on the interactive behavior of industry–university–research. The division of the dimensions and the findings of this study also further various related academic views (Li and Liu, 2013; Xie et al., 2018; Qiu and Haugland, 2019). Thirdly, the mechanism of the impact of the interactive behavior on cooperative innovation performance is demonstrated, which provides micro evidence for better achieving the goals of industry–university–research cooperation.

### Practical enlightenment

Various conclusions were drawn in this study from the perspective of knowledge management. The central conclusion of this study shows that the interactive behavior should be strengthened in the process of industry–university–research cooperation. Firstly, enterprises should increase contact with relevant universities and research institutes and establish long-term and stable exchange and cooperation relationships. Enterprises should also make use of the innovation resources of universities and research institutes to improve their knowledge absorptive capacity and independent innovation power. Secondly, universities and research institutes should adhere to open scientific research and strengthen their ties with enterprises to actively engage in industry–university–research cooperation. They should also strengthen the construction of the platforms for the integration of industry–university–research, and make full use of enterprise resources to promote teaching and scientific research. Most importantly, it is necessary to improve and perfect the incentive system for industry–university–research cooperation, and deepen the relationship of cooperative trust. Thirdly, the government should establish a combined online and offline information platform to promote the exchange of information on industry–university–research cooperation. It should improve and perfect the policy system of industry–university–research cooperation, and create a fair, honest, and inclusive market environment. The above recommendations are valuable guidance for enterprises, universities, and research institutes, and for the government.

### Conclusion

On the basis of the interactive behavior from a microperspective, this study explores the influence of interactive behavior on cooperative innovation performance. Firstly, the concept and dimensions of the interaction behavior of industry–university–research cooperation are defined. The existing theories and study results were also used to construct a theoretical model. The questionnaires were conducted with the help of the local Science Technology Department. A total of 325 valid questionnaires were collected. The study found that the interactive behavior of industry–university–research cooperation promotes the improvement of cooperative innovation performance. Specifically, cooperative communication and cooperative trust all play a role in promoting cooperative innovation performance. Knowledge absorptive capacity plays a partial role in mediating the relationship between interactive behavior and cooperative innovation performance. In the heterogeneity analysis, the effect of cooperative trust on cooperative innovation performance was not significant among low-size enterprises and was significant among large-size enterprises. Secondly, the effect of cooperative trust on cooperative innovation performance was significant among the “Low-Group” and not significant among the “High-Group.” Thirdly, the promoting effect of cooperation trust on cooperative innovation performance in the “Experienced group” was far greater than that in the “inexperienced group.” Future research can further explore the heterogeneity analysis of cooperative innovation performance.

### Final remarks

On the basis of the process of industry–university–research cooperation, it would be a valuable research topic to examine the influence of the interactive behavior on the cooperative innovation performance of industry–university–research cooperation, and to propose improvement countermeasures from the microbehavioral level of the subjects of industry–university–research cooperation. Despite its strengths, the study has certain deficiencies. Firstly, we only selected data from Zhejiang Province for the study, which is geographically limited. Subsequent studies can explore national data and analyze the heterogeneity of geographical areas. Secondly, the factors that influence the interactive behavior of industry–university–research are complex. Future research should further explore the antecedents and post-variables of interactive behavior, and more micro-influencing factors of cooperative innovation performance.

## Data availability statement

The original contributions presented in the study are included in the article/supplementary material, further inquiries can be directed to the corresponding author.

## Author contributions

XM and KD: conceptualization. XM: methodology, formal analysis, and writing—original draft preparation. HS: software. XJ and XH: validation. CW and LF: investigation and data curation. WL: resources. XM: writing—review and editing. All authors contributed to the article and approved the submitted version.

## Funding

This research was funded by Zhejiang Provincial Philosophy and Social Science Planning Project "Research on Improving the Competitiveness of Zhejiang Service Outsourcing Park under the Perspective of Sharing Economy, grant number 18NDJC311YBM.

## Conflict of interest

The authors declare that the research was conducted in the absence of any commercial or financial relationships that could be construed as a potential conflict of interest.

## Publisher’s note

All claims expressed in this article are solely those of the authors and do not necessarily represent those of their affiliated organizations, or those of the publisher, the editors and the reviewers. Any product that may be evaluated in this article, or claim that may be made by its manufacturer, is not guaranteed or endorsed by the publisher.
